# Emodin Prevented Depression in Chronic Unpredicted Mild Stress-Exposed Rats by Targeting miR-139-5p/5-Lipoxygenase

**DOI:** 10.3389/fcell.2021.696619

**Published:** 2021-07-26

**Authors:** Teng Zhang, Can Yang, Jiang Chu, Lin-Na Ning, Peng Zeng, Xiao-Ming Wang, Yan Shi, Bao-Jian Qin, Na Qu, Qi Zhang, Qing Tian

**Affiliations:** ^1^Department of Pathology and Pathophysiology, Key Laboratory of Neurological Disease of National Education Ministry, School of Basic Medicine, Tongji Medical College, Huazhong University of Science and Technology, Wuhan, China; ^2^Department of Neurology, Shanxian Central Hospital, the Affiliated Huxi Hospital of Jining Medical College, Heze, China; ^3^Department of Pathology, Gannan Medical University Pingxiang Hospital, Pingxiang, China; ^4^Department of Psychological Trauma, Wuhan Mental Health Center, Tongji Medical College, Huazhong University of Science and Technology, Wuhan, China; ^5^Research Center for Psychological and Health Sciences, China University of Geosciences, Wuhan, China; ^6^Department of Psychiatry, Liyuan Hospital, Tongji Medical College, Huazhong University of Science and Technology, Wuhan, China

**Keywords:** emodin, anti-depression, miR-139-5p, glycogen synthase kinase 3β, nuclear factor erythroid 2-related factor 2

## Abstract

**Background:**

The use of medicinal plant ingredients is one of the goals of developing potential drugs for treating depression. Compelling evidence suggests that anti-inflammatory medicines may block the occurrence of depression. We studied the effect of a natural compound, emodin, on the development of psychosocial stress-induced depression and the underlying mechanisms.

**Methods:**

Chronic unpredicted mild stress (CUMS) for 7 weeks was performed to replicate psychosocial stress in rats. The sucrose preference test, force swimming test, and open field test were used to evaluate their behaviors. The differentially expressed proteins in the hippocampus were analyzed using proteomics. Nissl staining and Golgi staining were used to detect the loss of neurons and synapses, immunohistochemical staining was used to detect the activation of microglia, and the enzyme-linked immunosorbent assay was used to detect the levels of pro-inflammatory cytokines. Western blotting, immunofluorescence, and quantitative polymerase chain reaction were also performed.

**Results:**

Hippocampal inflammation with up-regulated 5-lipoxygenase (5-LO) was observed in the depressed rats after CUMS exposure. The upregulation of 5-LO was caused by decreased miR-139-5p. To observe the effect of emodin, we screened out depression-susceptible (DeS) rats during CUMS and treated them with emodin (80 mg/kg/day). Two weeks later, emodin prevented the depression behaviors in DeS rats along with a series of pathological changes in their hippocampi, such as loss of neurons and spines, microglial activation, increased interleukin-1β and tumor necrosis factor-α, and the activation of 5-LO. Furthermore, we demonstrated that emodin inhibited its excess inflammatory response, possibly by targeting miR-139-5p/5-LO and modulating glycogen synthase kinase 3β and nuclear factor erythroid 2-related factor 2.

**Conclusion:**

These results provide important evidence that emodin may be a candidate agent for the treatment of depression and established a key role of miR-139-5p/5-LO in the inflammation of depression.

## Introduction

Depression is a common psychiatric disease and one of the main causes of disability, with a wide array of symptoms that affect somatic, cognitive, affective, and social processes. It is characterized by low mood, sadness, insomnia, lack of interest in studies, work, life, and so on. As a leading cause of global burden, the main treatments for depression are drug and psychological interventions (Murray et al., 2013; Smith, 2014). While effective, one-third of the people accepting drug intervention do not respond to these antidepressants, and others do not experience complete remission or relapse due to numerous side effects of the chemical and synthetic drugs (Rush et al., 2006; Shelton et al., 2010). Therefore, the use of medicinal plant ingredients, which have many therapeutic benefits, is one of the goals of developing potential drugs for treating depression.

Psychosocial and environmental factors are risk factors for the development of depression (Hodes et al., 2015; Wohleb et al., 2016), and the important role of neuroinflammation has been highlighted by compelling clinical and preclinical evidence (Troubat et al., 2021). Clinical and rodent studies showed that exposure to repeated psychosocial and environmental stressors caused considerable immunological alterations, which included the accumulation of pro-inflammatory cytokines and a decrease in anti-inflammatory cytokines in the blood and brain (Müller et al., 2006; Howren et al., 2009; Kim et al., 2016). As the major cellular component of the innate immune system in the brain and the first line of defense, microglia plays a critical role in neuroinflammation (Prinz et al., 2019). Activated microglia releases pro-inflammatory cytokines, such as interleukin (IL)-1, IL-6, tumor necrosis factor-α (TNF-α), and nitric oxide, as well as anti-inflammatory cytokines, including IL-4 and IL-10. Acute psychological stressors in humans have been identified to continuously increase circulating inflammatory factors (Steptoe et al., 2007). In a Trier social stress test, elevated levels of the pro-inflammatory cytokines IL-6 and TNF-α were observed in healthy control subjects (Gaab et al., 2005). Chronic unpredicted mild stress (CUMS), an experimental method of replicating psychosocial and environmental stressors (Willner, 1997), has been shown to cause hippocampal microglial activation (Yue et al., 2017; Wang Y. L. et al., 2018). Activation of the NOD-like receptor protein 3 inflammasome and upregulation of pro-inflammatory cytokines were observed in the hippocampus of depressed rats after CUMS (Yue et al., 2017; Wang Y. L. et al., 2018). These data suggested that neuroinflammation was an important mechanism that linked psychosocial stress to depression.

Thus, targeting neuroinflammation has been recognized as a potential strategy for the prevention of psychosocial and environmental stress-induced depression. Some clinical studies have indicated better antidepressant effects for anti-inflammatory drugs, especially non-steroidal anti-inflammatory drugs (NSAIDs) and cytokine-inhibitors (Kohler et al., 2016). However, some reported side effects have also raised controversy regarding the safe use of NSAIDs (Kohler et al., 2016). Emodin, a natural active compound extracted from the herb *rhubarb*, has anti-inflammatory and neuroprotective properties (Huang et al., 1992; Dong et al., 2016). In CUMS exposed mice, emodin reversed the behavioral deficiency, decreased the plasma corticosterone level, and up-regulated the mRNA and protein levels of hippocampal glucocorticoid receptor (GR) and mRNA levels of brain-derived neurotrophic factor (BDNF) (Li et al., 2014). Emodin has also shown protective in Alzheimeris disease (AD) mice (Wang et al., 2020) and stroke models (Leung et al., 2020; Liu Y. et al., 2020). These studies prompted that emodin may prevent depression through its anti-inflammatory properties.

Depression is associated with the alterations in regional brain volumes and functions, particularly the hippocampus (Otte et al., 2016). In this study, we evaluated the hippocampal inflammation and depression-like behaviors in rats exposed to CUMS. It was also tested whether emodin prevents the hippocampal inflammation in rats under CUMS exposure. Hippocampal inflammation with the activation of 5-lipoxygenase (5-LO) was observed in the rats with depression-like behaviors. To observe the effect of emodin, during 7 weeks of CUMS exposure, we screened out depression-susceptible (DeS) rats and stress-insensitive (Ins) rats at the end of the fifth week. DeS rats received emodin (Emo, 80 mg/kg/day). Two weeks later, the depression-like behaviors of emodin-treated DeS rats had improved. A series of pathological changes in the hippocampus, such as hippocampal neuron and spine loss, microglial activation, increased IL-1β and TNF-α, and the activation of 5-LO were revised by emodin. Additionally, it was also suggested that emodin played its protections by targeting miR-139-5p/5-LO.

## Materials and Methods

### Antibodies and Drugs

The primary antibodies used in this study are listed in Table 1. Emodin was obtained from a Shanghai Based Industry (Shanghai, China) and dissolved in Tween-80, a non-ionic surfactant and emulsifier used in foods (Sinopharm Chemical Reagent Co., Ltd., Beijing, China). Anti-rabbit or anti-mouse IgG conjugated to IRDye^@^ (800CW) (1:10,000) was purchased from Lincoln (United States). Toluidine blue and dimethyl sulfoxide were purchased from Sigma-Aldrich (St. Louis, MO, United States). The diaminobenzidine tetrachloride system was obtained from Beijing Zhongshan Jinqiao Biotechnology Co., Ltd. (Beijing, China). The miR-139-5p inhibitor was purchased from RiboBio Co., Ltd. (Guangzhou, China). The 3′-UTR of *5-LO* was amplified with the following primers: forward 5′-CGGGGTCTACAGTGCACGT-3′, reverse 5′-CTCAACTGGTGTCGTGGAGTC-3′.

**TABLE 1:** Antibodies used in this study.

Antibody	Epitopes	mAb/pAb	WB	IHC or IF	Source
α-tubulin	α-tubulin	mAb	1:2000		Abcam
Iba1	ionized calcium binding adapter molecule 1	pAb		1:200	Wako
t-GSK3β	total GSK3β	pAb	1:1000		Cell Signaling
p-GSK3β	p-GSK3β at Ser9	pAb	1:1000		Cell Signaling
t-Nrf2	total Nrf2	pAb	1:500		Abcam
p-Nrf2	p-Nrf2 at Ser40	pAb	1:1000	1:100	Abcam
5-LO	total 5-lipoxygenase	mAb	1:1000	1:100	Abcam
NF-κB p65	total NF-κB p65	mAb	1:500		Cell Signaling
GAPDH	full length GAPDH	mAb	1:1000		Abcam
Histone3	total histone H3 protein	pAb	1:1000		Cell Signaling

*p, phosphorylated; mAb, monoclonal antibody; pAb, polyclonal antibody; WB, Western blotting; IHC, immunohistochemistry; IF, immunofluorescence.*

### Cell Culture and Transfection

N2a cells were cultured with 45% DMEM-high glucose medium and 45% Opti-MEM^®^ I Reduced Serum Medium and supplemented with 10% fetal bovine serum, 100 U/ml penicillin, and 0.1 mg/ml streptomycin (all from HyClone) at 37°C in the presence of 5% CO_2_. Transfection was performed with neofect (Neofect Biotechnology, Beijing, China) when cells were cultured to 70–80% confluence in six-well plates. 48 h after transfection, the cells were collected and lysed for further research. HEK293 cells were cultured in high-glucose DMEM supplemented with 10% FBS, 100 U/ml penicillin, and 0.1 mg/ml streptomycin (all from HyClone). The cells were incubated at 37°C in a humidified atmosphere containing 5% CO_2_. HEK293 cells were transfected using Lipofectamine 2000 (Invitrogen, Carlsbad, CA, United States).

### Animals

8-week-old male Sprague-Dawley rats were supplied by the Experimental Animal Center of Tongji Medical College, Huazhong University of Science and Technology (Wuhan, China). All efforts were made to minimize animal suffering and to reduce the number of rats used. All experimental procedures in this study were approved by the Animal Care and Use Committee of Huazhong University of Science and Technology. Rats were housed five per cage in temperature-controlled rooms (26 ± 2°C) with standard rodent chow and water available *ad libitum*, kept on a standard 12 h light and dark cycle with the light on from 7:00 a.m. to 7:00 p.m. All rats were evaluated using the sucrose preference test (SPT), forced swimming test (FST), and open field test (OFT) before the experiments. The behavioral tests were performed during the light cycle in a dedicated sound-proof behavioral facility by experimenters blinded to the treatment information. Rats were brought to the procedure room 1 h before the start of the behavioral test and remained in the same room throughout the test. At all times, the sound was masked with a 60–65 dB white noise.

**FIGURE 1:**
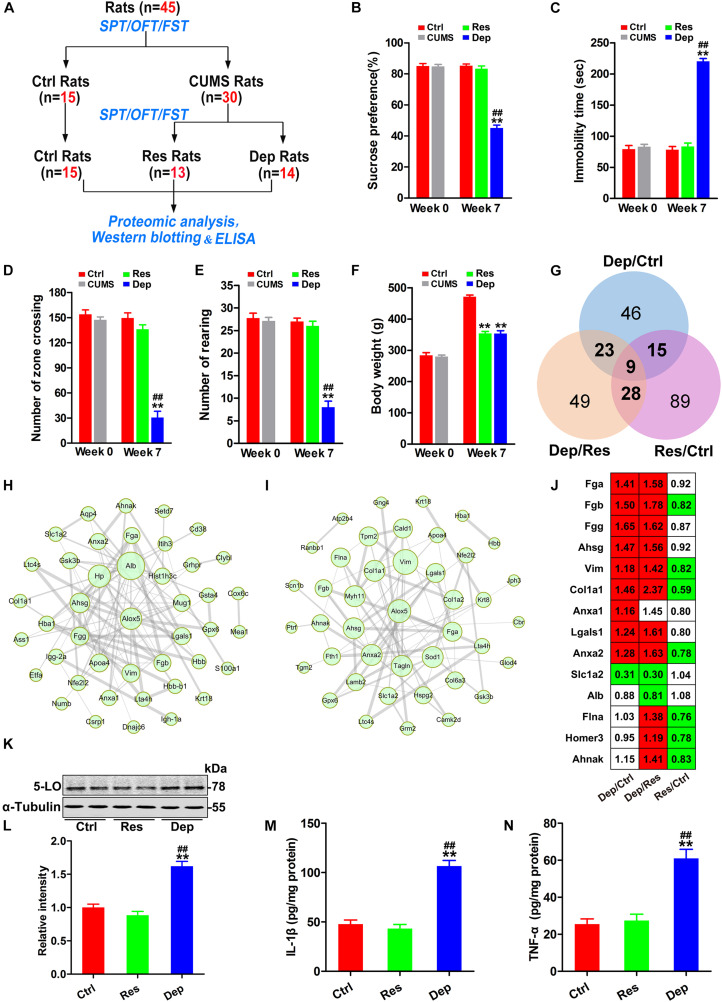
Inflammation with increased 5-lipoxygenase (5-LO) was observed in the hippocampi of Dep rats. Schematic illustration of the first part of this research **(A)**. Forty-five rats were evaluated by the sucrose preference test (SPT), forced swimming test (FST), and open field test (OFT). Then, 15 rats were randomly chosen as control (Ctrl) rats, and 30 rats were exposed to chronic unpredicted mild stress (CUMS). Seven weeks later, 13 depression resistant (Res) rats and 14 depression (Dep) rats were obtained. The hippocampi of rats were analyzed by proteomic analysis (*n* = 3/group), Western blotting (*n* = 6/group) and ELISA (*n* = 3/group). The sucrose preference rates in the SPT **(B)** [*F*_(__2_,_41__)_ = 128.332, *p* = 0.000], immobility time in the FST **(C)** [*F*_(__2_,_41__)_ = 160.933, *p* = 0.000], numbers of zone crossing **(D)** [*F*_(__2_,_41__)_ = 46.218, *p* = 0.000] and rearing times **(E)** [*F*_(__2_,_41__)_ = 57.185, *p* = 0.000] in the OFT, and the body weights of rats **(F)** [*F*_(__2_,_41__)_ = 127.246, *p* = 0.000] were recorded. Based on the identified differentially expressed proteins (DEPs) **(G)**, protein–protein interaction (PPI) networks of the DEPs in Dep/Ctrl rats **(H)** and Dep/Res rats **(I)** were constructed. The thickness of edges was decided by the combined score. The DEPs related to inflammation were listed with a ratio (**J**, red color means significant up-regulation and green color means significant down-regulation). Hippocampal levels of 5-lipoxygenase (5-LO) **(K,L)** [*F*_(__2_,_15__)_ = 42.049, *p* = 0.000], interleukin-1β (IL-1β, **M**) [*F*_(__2_,_15__)_ = 53.947, *p* = 0.000] and tumor necrosis factor-α (TNF-α, **N**) [*F*_(__2_,_15__)_ = 27.079, *p* = 0.000] in the Ctrl, Res, and Dep rats were tested by Western blotting **(K,L)** and ELISA **(M,N)**. Data were analyzed by a one-way ANOVA with LSD *post hoc* test and expressed as the means ± S.E.M. **p < 0.01 Res or Dep versus Ctrl. ^##^*p* < 0.01 Dep versus Res.

In the first part of this study (Figure 1A), 45 rats were used, of which 15 rats were randomly chosen as control (Ctrl) rats, and 30 rats were exposed daily for 7 weeks of CUMS as reported previously (Ning et al., 2018; Qu et al., 2020). Depressed (Dep) rats were defined as those whose sucrose water intake decreased by more than 30% in the SPT, and their resting time increased by 50% during the FST. Depression resistant (Res) rats were defined as those who had a greater sucrose water intake than the lower endpoint of the 95% confidence interval of the control rats in the SPT and a shorter immobility time than the upper endpoint of the 95% confidence interval of the control rats in the FST. After 7 weeks of CUMS exposure, 13 rats were classed into the Res group and 14 rats into the Dep group, and remaining three rats did not fit into either group (Figure 1A).

In the second part (Figure 3A), we screened 30 depression-susceptible rats (DeS) at the end of fifth week of CUMS exposure for 7 weeks from 64 CUMS-exposed rats. DeS rats was defined as those who had a >20% decrease in sucrose water intake in the SPT. Then, we treated control (Ctrl) and DeS rats with emodin (intragastric administration, 80 mg/kg) or the same volume of solvent (Veh) daily (Figure 3A). The dosage of emodin was determined from previous studies (Li et al., 2014; Zeng et al., 2019).

### Chronic Unpredicted Mild Stress (CUMS)

CUMS exposure for 7 weeks was performed as previously described (Ning et al., 2018; Qu et al., 2020). Briefly, all stress-exposed rats were subjected to three or four stressors each day, such as water or food deprivation for 24 h, empty water bottles for 2 h, cold room (4°C) for 2 h, hot room (45°C) for 15 min, cage tilt for 16 h, continuous overnight lighting for 12 h, soiled cage (200 ml of water spilled onto the bedding) for 12 h, grouped housing in one cage (4-5 per cage) for 12 h, strobe lighting (200 flashes/minute) for 4 h, and intermittent white noise (85 dB) for 6 h. The procedure was repeated for 7 weeks. The Ctrl rats were left undisturbed, with the exception of general handling (i.e., regular cage cleaning, water or food deprivation for the SPT, and measuring body weight). SPT and weight weighing were performed weekly.

### Sucrose Preference Test (SPT)

Sucrose preference test consisted of 7 days of training phase, 24 h of food and water deprivation phase, and 1 h of testing phase. As previously described, we trained the rats to consume 1.5% water sucrose solution for 1 h (9:30–10:30 am) every day to adapt to the novelty of the training phase. Then, the rats were deprived of food and water for 24 h. In the testing phase, rats were allowed free access to two pre-weighed bottles (containing water or 1.5% sucrose solution) for 1 h. Sucrose preference was calculated as a percentage of sucrose consumption × 100/(water consumption + sucrose consumption).

### Open Field Test (OFT)

The test was performed in a bare square box 100 cm in length, 100 cm in width, and 40 cm in height. As previously described (Ning et al., 2018), each rat was placed in the center of a black floor with 25 equal squares (20 cm × 20 cm square), including 16 peripheral squares and nine central squares. The activity of the rats was recorded using an overhanging camera linked to a computer over a 5 min’ period. The number of total squares a rat crossing in the arena defined as the number of zones crossing, was analyzed as measures of locomotor activity. The rearings were taken as measures of anxiety. The box was cleaned with 75% alcohol between tests.

### Forced Swimming Test (FST)

Next, the rats were tested in transparent Plexiglas cylinders (20 cm in diameter, 50 cm in depth, filled with 23–25°C water to a depth of 25 cm) as previously described (Qu et al., 2020). Rats were individually forced to swim for 5 min (Qu et al., 2020). Each session was videotaped for analysis, and the water was changed between sessions. The duration of immobility was measured for 5 min. Floating vertically in the water and making only those movements necessary to maintain the head above the surface of the water for living were both considered as immobility. Immobility time was used to assess feelings of hopelessness in rats.

### Proteomic Analysis

Hippocampal proteomic analyses were conducted as previously described (Fang et al., 2018; Ning et al., 2018; Qu et al., 2020). Briefly, the hippocampal proteins were extracted, digested, and labeled with iTRAQ-6plex reagents in accordance with the manufacturer’s protocol. The peptides were then fractionated by high-pH reverse-phase HPLC. The resulting fractions were dissolved, loaded onto a reversed-phase pre-column (Acclaim PepMap 100, Thermo Scientific), and then separated using a reversed-phase analytical column (Acclaim PepMap RSLC, Thermo Scientific). The peptides were subjected to NSI source followed by tandem mass spectrometry (MS/MS) in Q Exactive^*TM*^ Plus (Thermo) coupled online to the UPLC. In order to identify the proteins, we analyzed the resulting MS/MS data using the Mascot search engine (v.2.3.0) and searched against the Uniprot_rat database (32,983 sequences). We defined proteins with isobaric tags for relative and absolute quantitation (iTRAQ) ratios of >1.15, or <0.87, coupled with *p* < 0.05 as the differentially expressed proteins. The interaction network of the differentially expressed proteins was constructed using STRING 11.0.

### Brain Slice Preparation

Rats were anesthetized with isoflurane and transcardially perfused with 100 mL normal saline and then perfused with 400 mL of 4% paraformaldehyde solution. The brains were carefully removed from the skull. For Nissl staining, immunohistochemical staining, and immunofluorescence staining, the brains were post-fixed in 4% paraformaldehyde solution for another 24 h at 4°C. During dehydration, the samples were subjected to 20 and 30% sucrose gradient dehydration twice until they were completely sunken. All brains were sliced into 30 μm coronal sections with a freezing microtome (Kryostat, 1720, Leitz, Wetzler, Germany). The sections were consecutively collected and stored in 50% glycerin in PBS at −20°C.

### Nissl Staining

Nissl staining was conducted as previously described (Qu et al., 2020). The sections were washed with PBS for 2 min × 3 times, pasted on the slides, and air-dried for 2 h. They were then immersed in Nissl dye liquor for several minutes according to the color change, followed by decoloration in 75% alcohol and 95% alcohol twice for several minutes each. Subsequently, absolute ethyl alcohol was used for dehydration for 5 min × 3 times and dimethylbenzene for transparency for 10 min twice. The sections were sealed and dried in a fume hood. Images were obtained using an optical microscope (Nikon 90i, Tokyo, Japan).

### Immunohistochemical and Immunofluorescence Staining

After being washed with PBS for 5 min × 3 times and treated with PBS containing 0.3% H_2_O_2_ and 0.5% Triton X-100 for 30 min at room temperature, the sections were pre-incubated with 3% normal goat serum and incubated with the primary antibodies (Table 1). After 24 h, the cells were incubated for 1 h with the secondary antibodies (Table 1) for 1 h at 37°C after being washed with PBS. For immunohistochemical staining, immunoreaction was developed using Histostain TM-SP kits (ZSGB-Bio, Beijing, China) and visualized with diaminobenzidine (brown). Images were obtained using an optical microscope (Nikon 90i, Tokyo, Japan). For immunofluorescence-stained sections, the images were observed using a laser scanning confocal microscope (Zeiss LSM 710, Germany).

### Golgi Staining

Golgi staining was conducted as previously described (Qu et al., 2016). After the brain was removed from the skull, Golgi staining was performed using the FD Rapid GolgiStain kit (FD Neurotechnologies, Baltimore, MD, United States) according to the manufacturer’s protocol. The samples were cut into horizontal sections of 100 μm thickness using a vibratome (Leica, Nussloch, Germany; S100, TPI) and mounted on gelatin-coated slides. The sections were dehydrated in successive alcohol and transparent in xylene, and then sealed. The sections were observed and imaged using an ordinary optical microscope (Nikon 90i, Tokyo, Japan). The number of dendritic spines on hippocampal pyramidal neurons was counted using Image-Pro Plus 6.0 software (Media Cybernetics, Inc., United States).

### Western Blotting

The rats were anesthetized with isoflurane and sacrificed. The hippocampi were quickly dissected out of the brain and frozen at −80°C. For the analysis of whole cell components, the sample of hippocampus was homogenized in cold buffer solution containing 10 mM Tris-HCl (pH 7.4), 50 mM NaCl, 50 mM NaF, 0.5 mM Na_3_VO_4_, 1 mM EDTA, 1 mM benzamidine, 1.0 mM phenylmethylsulfonyl fluoride (PMSF), 5 mg/ml leupeptin, 5 mg/ml aprotinin, and 2 mg/ml pepstatin. Lysates were mixed with 4 × extraction buffer, and protein concentrations were determined using a BCA protein assay kit (Rockford, IL, United States). All sample solutions were stored at −80°C until use. For western blotting of nuclear and cytoplasmic fractions (Qu et al., 2016), proteins were extracted with a kit from KeyGen Biotech (NanJing KeyGen Biotech Co., Ltd.).

Before sample loading, a final concentration of 10% β-mercaptoethanol and 0.05% bromophenol blue was added. The proteins were separated by 10% sodium dodecyl sulfate polyacrylamide gel electrophoresis (SDS-PAGE) and probed with primary antibodies (Table 1). Secondary antibodies were anti-rabbit or anti-mouse IgG conjugated to IRDye^@^ (800 CW; 1:10,000). The intensities of immunoblotting strips were automatically recognized by the Odyssey system (Li-Cor Bioscience, Lincoln, NE, United States). All intensities of the strips were normalized to the average intensity of α-tubulin. Then, the average value of the Ctrl group was taken as 1, and the relative intensity of each strip was calculated.

### Quantitative Real-Time Polymerase Chain Reaction (qPCR)

Total RNA was extracted from the hippocampus using TRIzol reagent (Invitrogen, Carlsbad, CA, United States) according to the manufacturer’s protocol. RNA_260__/__280_ was measured spectrophotometrically for determining the concentration and purity. To synthesize cDNA from miRNA, we used the M-MLV Reverse Transcriptase cDNA Synthesis Kit (Invitrogen, Carlsbad, CA, United States) according to the supplier’s recommendations. qPCR amplification was performed using the CFX96 Real-Time PCR Detection System (Bio-Rad, Hercules, CA, United States) and SYBR Green Premix Ex TaqTM (TaKaRa, Kyoto, Japan). The reaction conditions were as follows: initial denaturation for 3 min at 95°C, followed by 45 cycles of 10 s at 95°C, 30 s at 60°C, and extension for 30 s at 72°C. Primers used for qPCR analysis were designed and synthesized. Data were quantified using the ΔΔCt method.

### Luciferase Assays for Identifying miR-Target Interactions

Normal and mutated 3′-UTR sequences of *5-LO* were subcloned into the psiCHECK-2 reporter plasmid (Beijing Biotechnology Co., Ltd., Wuhan, China) as previously described (Wang X. et al., 2018). HEK293T cells were transfected with the psiCHECK-2 plasmid containing the 3′-UTR and the overexpression vector for a specific miRNA. 24 h after transfection, cells were lysed and luciferase reporter activity was assayed as previously described (Wang X. et al., 2018).

### Enzyme-Linked Immunosorbent Assay (ELISA)

The hippocampal levels of IL-1β, TNF-α, and leukotriene B4 (LTB4) were assayed by ELISA according to the protocols of ELISA kits (Elabscience Biotechnology Co., Ltd., Wuhan, China). A microplate reader (BioTek, Winooski, VT, United States) at 450 nm was used to determine the optical density (OD). A standard curve was created by plotting the mean OD values for each standard. The sample concentration was determined using a standard curve.

### Predictions of MicroRNA Targeting

The miR-targeting predictions were performed using three different web-based algorithms, TargetScan^1^, miRbase^2^, and miRDB^3^.

### Statistical Analysis

The data were analyzed using SPSS (version 12.0; SPSS Inc., Chicago, IL, United States) and statistical graphs were produced using GraphPad Prism (GraphPad Software, Inc., La Jolla, CA, United States) and expressed as mean ± S.E.M. Differences among the groups were tested using an one-way analysis of variance (ANOVA) and LSD *post hoc* test. The two-way

ANOVA and Bonferroni’s *post hoc* test compared the differences between groups that have been split on two independent factors. The level of significance was set at *p* < 0.05.

## Results

### Inflammation With Increased 5-LO Was Found in the Hippocampus of Dep Rats

Before CUMS exposure, all the rats (*n* = 45) had identical rates of preference for sucrose (approximately 85%) in the SPT, equivalent immobility time (about 79.3 s) in the FST, and equivalent number of zone crossing (about 154) and rearing times (about 27) in the OFT. After 7 weeks of CUMS exposure, 13 Res rats and 14 Dep rats were obtained (Figure 1A). The Dep rats showed a robust decrease (45.1%) in sucrose intake, whereas Res rats displayed a sucrose preference (83.2%) similar to that of unstressed Ctrl rats (Figure 1B). In the FST, the Dep rats had a much longer immobility time (220 ± 4.4 s) than Res rats (83.3 ± 5.8 s) and Ctrl rats (78.1 ± 5.3 s) (Figure 1C). In the OFT, the number of zone crossings and rearing times of Dep rats were lower than those of Res rats and Ctrl rats (Figures 1D,E). Additionally, we found that both Dep (354.1 ± 6.4 g) and Res (353.5 ± 9.5 g) rats had lighter body weights than Ctrl rats (471.1 ± 5.8 g) (Figure 1F).

In the proteomics data with 3645 quantified proteins, 262 differentially expressed proteins were identified, which included 93 differentially expressed proteins in Dep/Ctrl, 109 in Dep/Res, and 141 in Res/Ctrl (Figure 1G). Protein-protein interaction (PPI) network analysis suggested that inflammation was the characteristic signal in the hippocampus of Dep rats (Figures 1H,I). Increased fibrinogen a (Fga), Fgb, Fgg, and α2-HS glycoprotein (Ahsg) were observed in Dep/Ctrl and Dep/Res, but not in Res/Ctrl rats (Figure 1J), suggesting hippocampal inflammation in the development of depression. Additionally, increased vimentin (Vim) (Huang et al., 2016), collagen type I α 1 chain (Col1a1) (Amantea et al., 2009), annexin A1 (Anxa1) (Zub et al., 2019), Anxa2 (Tu et al., 2019), and Galectin-1 (Gal-1, lgals1) (Sundblad et al., 2017) indicated elevated inflammatory responses to CUMS and/or increased the permeability of the blood–brain barrier (BBB) in the hippocampus (Huang et al., 2016) (Figure 1J). Astrocytic glutamate transporter-1 (GLT1, slc1a2) is responsible for up to 95% of extracellular glutamate clearance and is essential for brain function (Pregnolato et al., 2019). Pro-inflammatory cytokines have been shown to significantly down-regulate slc1a2 (Lee et al., 2017). As slc1a2 was down-regulated in Dep/Ctrl and Dep/Res rats (Figure 1J), we tested the levels of pro-inflammatory cytokines IL-1β and TNF-α in the hippocampi by ELISA. The levels of IL-1β and TNF-α in Dep rats were more than twice as high as those in Res and Ctrl rats (Figures 1M,N). In the PPI network analysis, 5-LO (ALOX5) was shown to have more connections with the differentially expressed proteins in Dep/Ctrl (Figure 1H) and Dep/Res rats (Figure 1I). Western blotting confirmed that the level of 5-LO in the hippocampus of Dep rats was much higher than that of Res and Ctrl rats (Figures 1K,L). The data highlighted the important role of hippocampal inflammation, with 5-LO increasing in the development of depression.

### Reduction of miR-139-5p Induced 5-LO Elevation

**FIGURE 2:**
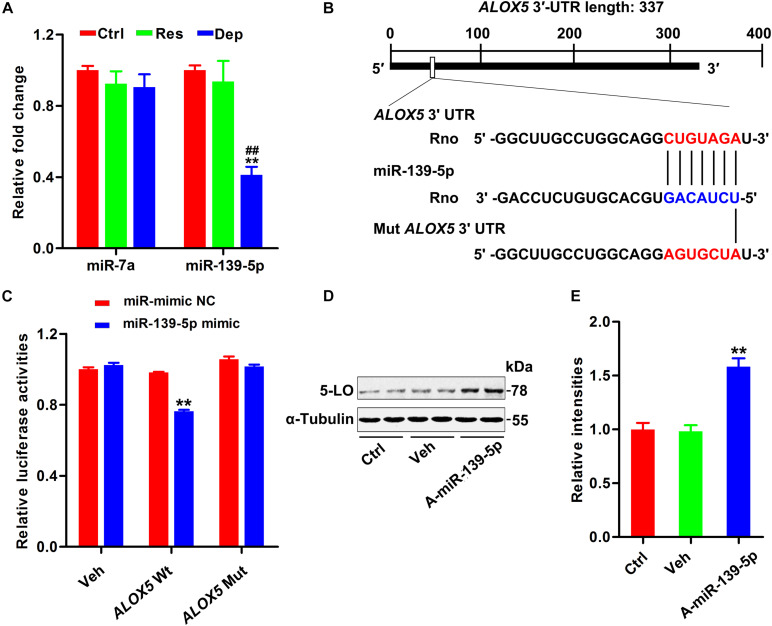
Downregulation of miR-139-5p was responsible for 5-LO elevation. Alterations in predicted miRNAs that target 5-LO in the hippocampus of CUMS-exposed rats (**A**, *n* = 3 brains/group) [*F*_(__2_,_15__)_ = 0.717 for miR-7a, *p* = 0.504; *F*_(__2_,_15__)_ = 19.131 for miR-139-5p, *p* = 0.000]. **p < 0.01 Res or Dep versus Ctrl. ^##^*p* < 0.01 Dep versus Res, one-way ANOVA with LSD *post hoc* test. The binding site for miR-139-5p seed sequence in the *ALOX5* 3′-UTR **(B)**. Luciferase reporter assay results demonstrated that miR-139-5p targeted *ALOX5* 3′-UTR **(C)** [*F*_(__1_,_20__)_ = 145.831 for *ALOX5* wt, *p* = 0.000; *F*_(__1_,_20__)_ = 1.275 for *ALOX5* mut, *p* = 0.272]. **p < 0.01 miR-139-5p mimic versus the negative control-treated group (miR mimic NC), two-way ANOVA and Bonferroni’s *post hoc* test. The 5-LO level in N2a cells treated with miR-139-5p inhibitor (A-miR-139-5p) or its scrambled control (Veh0) was tested by western blotting **(D)** and quantitatively analyzed **(E)** [*F*_(__2_,_15__)_ = 27.071, *p* = 0.000]. **p < 0.01 A-miR-139-5p versus Veh, one-way ANOVA with LSD *post hoc* test.

Although 5-LO plays a key role in inflammation (Jatana et al., 2006; Joshi et al., 2014a; Liu C. H. et al., 2020), its regulation is still not fully understood. Using TargetScan software (see Text Footnote 1), miRbase (see Text Footnote 2), and miRDB (see Text Footnote 3), we found that miR-139-5p and miR-7a were scored the highest in both predicted outputs. By qPCR, the hippocampal level of miR-139-5p was found to be dramatically decreased in Dep rats, but not in Res rats (Figure 2A). To verify the post-transcriptional regulation of 5-LO by miR-139-5p, we constructed the wild-type 3′-UTR of 5-LO (*ALOX5* Wt, containing the binding site to miR139-5p) and a mutated version (*ALOX5* Mut) to the luciferase reporter vector, which were co-transfected into HEK293 cells with miR-139-5p mimic or a negative control (miR-mimic NC) (Figure 2B). Overexpression of miR-139-5p suppressed the expression of the luciferase reporter gene with the *5-LO* 3′-UTR, whereas the expression of the reporter was not suppressed when we mutated the miR-139-5p recognition site located on the *5-LO* -3′ UTR (Figure 2C). Moreover, the miR-139-5p inhibitor elevated the protein level of 5-LO in N2a cells (Figures 2D,E). These results showed that miR-139-5p inhibited 5-LO expression at the post-transcriptional level, while the loss of miR-139-5p mediated 5-LO elevation in depression.

### Emodin Ameliorated Depression-Like Behaviors in DeS Rats

**FIGURE 3:**
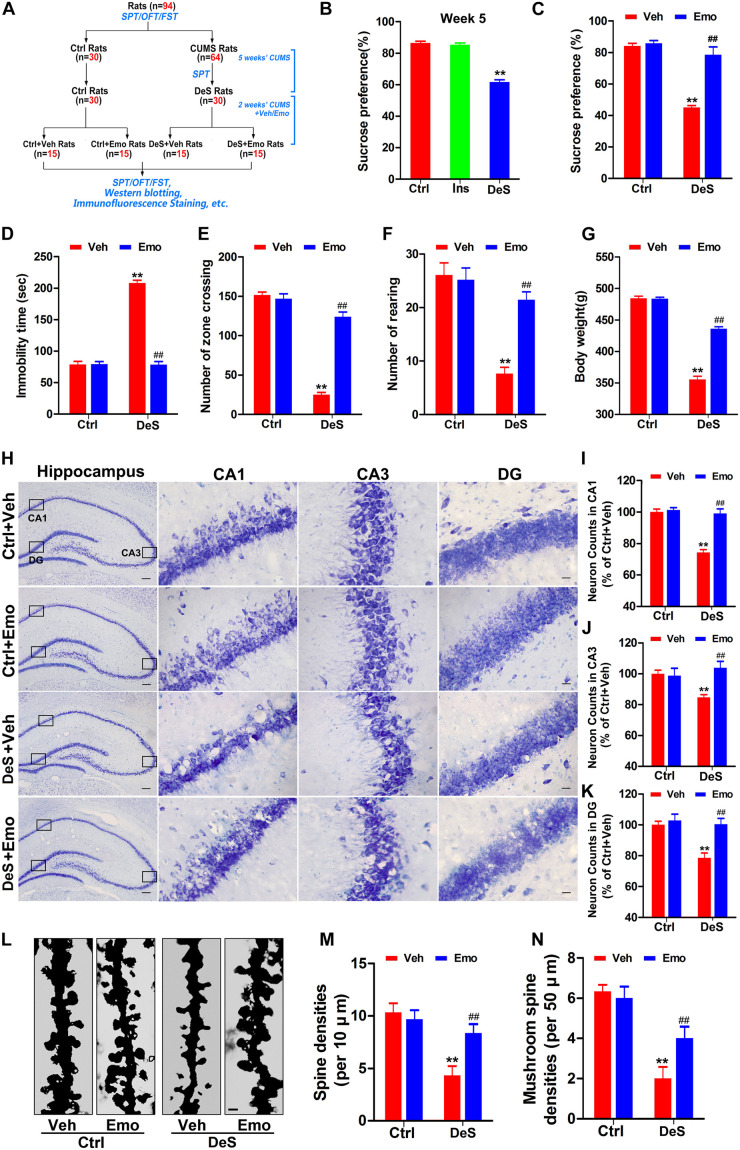
Emodin ameliorated depression-like behaviors in DeS rats. Schematic illustration of the second part of this research **(A)**. The sucrose preference test (SPT), forced swimming test (FST), and open field test (OFT) were performed as shown. By SPT, we selected 30 depression-susceptible rats (DeS rats) from 64 rats at the end of fifth week **(B)** [*F*_(__2_,_86__)_ = 143.894, *p* = 0.000]. 30 unstressed rats were the control (Ctrl). Then, the DeS and Ctrl rats accepted emodin (Emo) or the same volume of solvent (Veh) treatment. The Emo treated (DeS + Emo) and Veh treated DeS rats (DeS + Veh) were continuously exposed to CUMS. Finally, the sucrose preference rates in the SPT **(C)** [*F*_(__3_,_56__)_ = 48.668, *p* = 0.000], immobility time in the FST **(D)** [*F*_(__3_,_56__)_ = 255.939, *p* = 0.000], numbers of zone crossing **(E)** [*F*_(__3_,_56__)_ = 153.596, *p* = 0.000] and rearing times **(F)** [*F*_(__3_,_56__)_ = 25.595, *p* = 0.000] in the OFT, and the body weights **(G)** [*F*_(__3_,_56__)_ = 307.749, *p* = 0.000] were recorded (*n* = 15/group). Hippocampal neurons were shown by Nissl staining (**H**, left scale bar = 200 μm, right scale bar = 20 μm) and quantified in CA1 **(I)** [*F*_(__3_,_8__)_ = 36.35, *p* = 0.000], CA3 **(J)** [*F*_(__3_,_8__)_ = 10.681, *p* = 0.004], and DG **(K)** [*F*_(__3_,_8__)_ = 10.862, *p* = 0.003] regions (*n* = 3 brains/group). The dendrites of CA1 neurons were shown by Golgi staining (**L**, *n* = 3 brains/group, scale bar = 1 μm). Quantifications of the densities of spine **(M)** [*F*_(__3_,_8__)_ = 9.286, *p* = 0.006] and mushroom-type spine **(N)** [*F*_(__3_,_8__)_ = 14.500, *p* = 0.001] were calculated by Image-Pro Plus 6.0 software (*n* = 21 dendrites from 3 brains/group). Data were analyzed by one-way ANOVA with LSD *post hoc* test and presented as means ± S.E.M. **p < 0.01 DeS + Veh versus Ctrl + Veh, ^##^*p* < 0.01 DeS + Emo versus DeS + Veh.

We investigated whether emodin could prevent the development of depression in rats exposed to CUMS. In the first part of the study (Figure 1A), the success rate of depression replication in rats after 7 weeks of CUMS was 46.67%. Therefore, we selected 30 depression-susceptible rats (DeS rats) from 64 rats at the end of fifth week of CUMS exposure for 7 weeks. The DeS rats showed a significantly lower sucrose preference rate (61.6%) than the stress-insensitive rats (85.3%) and control rats (86.5%) (Figure 3B). Then, 30 DeS rats and 30 Ctrl rats received daily emodin treatment (Emo, 80 mg/kg) or the same volume of solvent treatment (Veh) for 2 weeks (Figure 3A). The DeS rats were continuously exposed to CUMS. Two weeks later, DeS + Veh rats (body weight 355.7 ± 5.0 g) presented depressive behaviors, such as a lower sucrose preference (44.1%) in the SPT (Figure 3C) and a longer immobility time (210.2 ± 3.4 s) in the FST (Figure 3D). However, DeS + Emo rats had a higher percent of sucrose preference (78.5%) in the SPT (Figure 3C) and a much shorter immobility time (78.5 ± 5.0 s) in the FST (Figure 3D), much more crossing zones and rearing times in the OFT (Figures 3E,F), and an increased body weight (435.6 ± 2.9 g, Figure 3G) than DeS + Veh rats. Emodin treatment had no effect on the emotional behavior and body weight of the Ctrl rats.

Studies in humans and animals have confirmed reduced volume of the hippocampus in depressed brains is characterized by the loss of neurons and synapses (Duman and Aghajanian, 2012; Kang et al., 2012). In this study, Nissl staining revealed that the DeS + Veh rats had fewer neurons in the hippocampal CA1, CA3, and DG regions (Figures 3H–K), whereas no obvious hippocampal neuron loss was observed in the DeS + Emo rats (Figures 3H–K). Golgi staining revealed a significant decrease in the density of dendritic spines and number of mushroom-type spines in CA1 (Figures 3L–N). DeS + Emo rats had more spines, especially mushroom-type spines, than the DeS + Veh rats (Figures 3L–N). Similar alterations in dendritic spines in CA3 and DG were observed (data not shown). Emodin treatment had no effect on hippocampal neuron number and spine density in the Ctrl rats. Data suggested that emodin prevented depression-like behaviors and ameliorated the loss of hippocampal neurons and dendritic spines in depressed brains.

### Emodin Inhibited the Activation of 5-LO by Upregulating miR-139-5p

**FIGURE 4:**
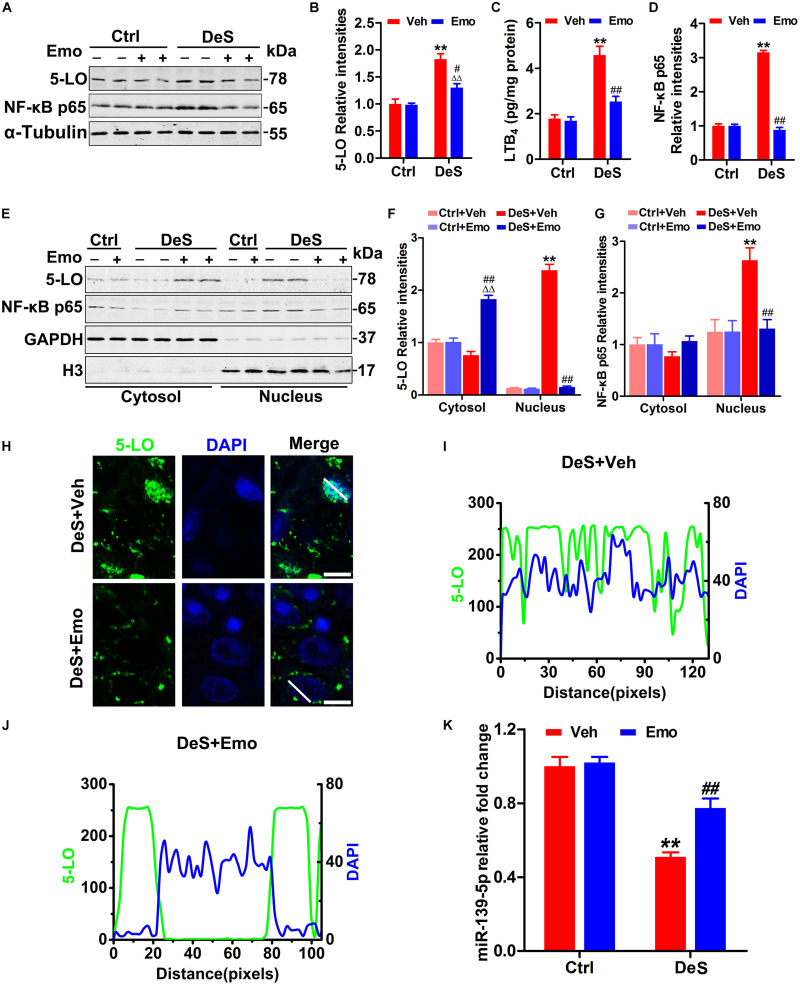
Emodin inhibited the activation of 5-LO and nuclear factor-κB (NF-κB) by up-regulating miR-139-5p. Hippocampal levels of 5-LO and activated NF-κB (NF-κB p65) were tested by Western blotting **(A)** and quantitative analysis **(B,D)** [*F*_(__3_,_20__)_ = 45.668 for 5-LO, *p* = 0.000; *F*_(__3_,_20__)_ = 541.762 for NF-κB, *p* = 0.000] (*n* = 6 brains/group). By ELISA, hippocampal levels of leukotriene B4 (LTB4) were assessed **(C)** [*F*_(__3_,_8__)_ = 26.844, *p* = 0.000] (*n* = 3 brains/group). The levels of 5-LO and NF-κB p65 in the nuclear and cytoplasmic fractions were detected by Western blotting **(E)** and quantitative analysis **(F,G)** (*n* = 6 brains/group). The loading amount of cytosolic samples was 10 μg and the loading amount of nuclear samples was 20 μg. By immunofluorescence staining (**H**, scale bar = 20 μm, *n* = 3 brains/group) and fluorescence intensities detection (**I,J**, the quantified areas were defined by the white lines), the positional relationships between 5-LO (green) and the nucleus (blue, DAPI) in CA1 neurons were shown. Hippocampal miR-139-5p was analyzed by qPCR **(K)** [*F*_(__3_,_8__)_ = 53.156, *p* = 0.000] (*n* = 3 brains/group). Data were analyzed by one-way ANOVA with LSD *post hoc* test and presented as means ± S.E.M. **p < 0.01 DeS + Veh versus Ctrl + Veh, ΔΔ*p* < 0.01 DeS + Emo versus Ctrl + Emo, ^#^*p* < 0.05, ^##^*p* < 0.01 DeS + Emo versus DeS + Veh.

5-LO, an important pro-inflammatory enzyme widely expressed in the brain, initiates leukotriene (LT) synthesis from arachidonic acid (Joshi et al., 2014b). DeS + Veh rats had higher levels of 5-LO and LTB4 (a major metabolic product of 5-LO activation) than DeS + Emo rats and Ctrl + Veh rats (Figures 4A–C). Emodin did not change the levels of 5-LO and LTB4 in the Ctrl rats (Figures 4A–C). Upon stimulation, 5-LO can be translated to nuclear and perinuclear membranes, and this translocation is regarded as a determinant of its LTB4 synthetic capacity (Luo et al., 2003). Therefore, the homogenate of the hippocampus was divided into cytoplasmic and nuclear fractions. DeS + Veh rats, but not DeS + Emo rats, had significantly higher nuclear 5-LO levels than the Ctrl + Veh and Ctrl + Emo rats (Figures 4E,F). Immunofluorescence staining and fluorescence intensity distribution analysis (FIDA) in neurons of the CA1 (Figures 4H–J), CA3, and DG (data not shown), more 5-LO was observed in the nucleus of the DeS + Veh rats, whereas it was mainly in the cytoplasm in the DeS + Emo (Figures 4H–J) and Ctrl rats (data not shown).

5-LO inhibition may down-regulate nuclear factor-κB (NF-κB) (Jatana et al., 2006; Yu and Chung, 2006). NF-κB, consisting of p50 and p65, resides in the cytoplasm of resting cells. In response to stimulation, NF-κB p65 is activated and translocated to the nucleus, followed by binding to specific DNA sequences in target genes involved in inflammation and apoptosis (Ahn and Aggarwal, 2005; Yu and Chung, 2006). In this study, increased NF-κB p65 was found in the hippocampi of the DeS + Veh rats (Figures 4A,D), and this increase was mainly concentrated in the nuclear fraction (Figures 4E,G). Emodin treatment significantly prevented the increase in nuclear NF-κB p65 levels (Figures 4E,G). We also found that hippocampal miR-139-5p was significantly lower in the DeS + Veh rats than in Ctrl + Veh rats (Figure 4K), whereas miR-139-5p was partially reduced in the DeS + Emo rats (Figure 4K). These results suggested that emodin partially inhibited the activation of 5-LO by upregulating miR-139-5p.

### Depression Associated Microglia Activation Was Inhibited by Emodin

**FIGURE 5:**
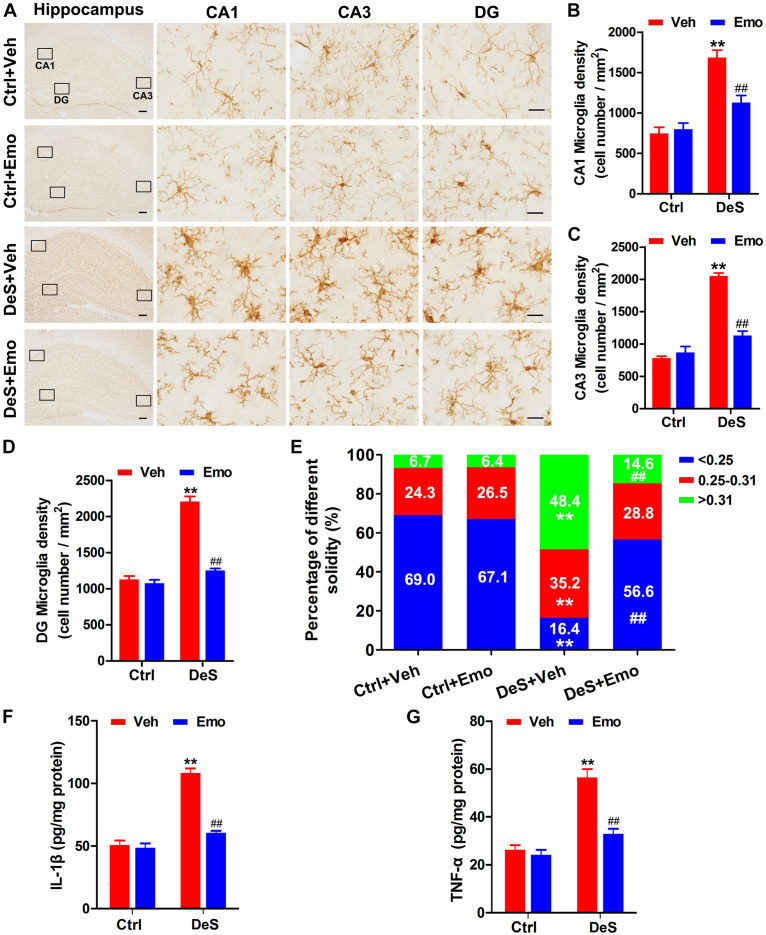
Depression associated microglia activation was inhibited by emodin. By immunohistochemistry staining with Iba1 (a marker of microglia), hippocampal microglia were shown **(A)** (left scale bar = 200 μm, right scale bar = 20 μm). The densities of microglia in CA1 **(B)** [*F*_(__3_,_8__)_ = 27.088, *p* = 0.000], CA3 **(C)** [*F*_(__3_,_8__)_ = 82.790, *p* = 0.000] and DG **(D)** [*F*_(__3_,_8__)_ = 13.656, *p* = 0.000] regions were calculated (*n* = 3 brains/group). The solidity value analysis was used to evaluate the activation of microglia. Higher solidity value indicated higher activity of microglia. The solidity values of the microglia were divided into three grades (<0.25, 0.25–0.31, >0.31), and the percentages of microglia with different grades in different groups were shown (**E**, *n* = 3 brains/group). By ELISA, hippocampal levels of interleukin-1β (IL-1β, **F**) [*F*_(__3_,_8__)_ = 34.317, *P* = 0.000] and tumor necrosis factor-α (TNF-α, **G**) [*F*_(__3_,_8__)_ = 22.465, *P* = 0.000] were assessed (*n* = 3 brains/group). Data were analyzed by one-way ANOVA with LSD *post hoc* test and presented as means ± S.E.M. **p < 0.01 DeS + Veh versus Ctrl + Veh, ^##^*p* < 0.01 DeS + Emo versus DeS + Veh.

Microglia are associated with inflammation in depression (Kreisel et al., 2014; Yirmiya et al., 2015). By Iba1 (a microglial marker)-based immunohistochemical staining (Figure 5A), the densities of microglia in the hippocampal CA1, CA3, and DG regions of the DeS + Veh rats were determined. The densities of microglia in the DeS + Emo rats were significantly lower than those in DeS + Veh rats (Figures 5A–D). Increased solidity (the ratio of the positive area to the convex area) represents the activation of microglia (Soltys et al., 2001; Yang et al., 2016; Zeng et al., 2019). In this research, we divided the solidity value of the microglia into three grades, which were <0.25 (ramified), 0.25–0.31 (hypertrophied) and >0.31 (bushy, also termed as amoeboid), and a higher solidity value indicated higher activation of microglia (Soltys et al., 2001). As shown in Figure 5E, 69% of microglia in the Ctrl + Veh rats, 67.1% in the Ctrl + Emo rats, and 56.6% in the DeS + Emo rats had solidity values below 0.25. Only 16.4% of microglia in DeS + Veh rats had a solidity value below 0.25. DeS + Veh rats had the most microglia (48.4%) with the highest solidity value, greater than 0.31, which indicated their activation. ELISA showed that the DeS + Veh rats had significantly increased levels of IL-1β and TNF-α in their hippocampi (Figures 5F,G). There was no difference in the proportion of microglia solidity values and levels of IL-1β and TNF-α between the DeS + Emo and Ctrl rats. Data suggested that depression-associated microglial activation was inhibited by emodin.

### Emodin Inhibited Hippocampal GSK3β Activation

**FIGURE 6:**
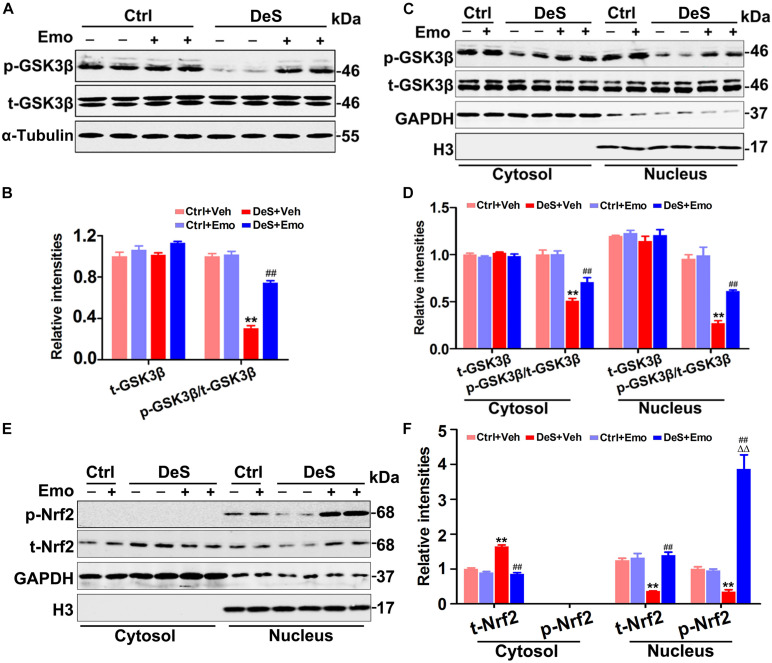
Emodin inhibited hippocampal glycogen synthase kinase 3β (GSK3β) activation. The levels of total GSK3β (t-GSK3β) and phosphorylated GSK3β (p-GSK3β, Ser9) were shown by Western blotting **(A)** and quantitatively analyzed **(B)** [*F*_(__3_,_20__)_ = 90.46 for p-GSK3β/t-GSK3β, *p* = 0.000] (*n* = 6 brains/group). Meanwhile, the levels of t-GSK3β, p-GSK3β and total nuclear factor erythroid 2-related factor 2 (t-Nrf2) in the nuclear and cytoplasmic fractions were also tested by Western blotting **(C,E)** and quantitatively analyzed (**D,F**, *n* = 6 brains/group). The loading amount of cytosolic samples was 10 μg and the loading amount of nuclear samples was 20 μg. Data were analyzed by one-way ANOVA with LSD *post hoc* test and presented as means ± S.E.M. **p < 0.01 DeS + Veh versus Ctrl + Veh, ^ΔΔ^*p* < 0.01 DeS + Emo versus Ctrl + Emo, ^##^*p* < 0.01 DeS + Emo versus DeS + Veh.

GSK3β is suggested to be involved in the pathogenesis of depression, and is a target and/or modifier of antidepressant action (Duda et al., 2020). Previous research suggested that stress led to GSK3β activation depending on 5-LO (Joshi et al., 2014b). In this study, the total levels of hippocampal GSK3β were equal in all groups, while its phosphorylation level at Ser9 (p-GSK3β, inactive form) was significantly decreased in the DeS + Veh rats (Figures 6A,B). Furthermore, a decrease in phosphorylated GSK3β at Ser9 was observed in both the cytoplasmic and nuclear fractions of the DeS + Veh rats (Figures 6C,D). In the hippocampi of DeS + Emo rats, the phosphorylation levels of GSK3β at Ser9 in both cytoplasmic and nuclear fractions were higher than those in the DeS + Veh rats, which indicated the inhibition of GSK3β activation by emodin (Figures 6C,D). Nuclear factor erythroid 2-related factor 2 (Nrf2) is a transcriptional activator of antioxidant genes and is down-regulated in depression (Bansal et al., 2019). GSK3β has been reported to directly phosphorylate Nrf2 and shift the subcellular distribution of Nrf2 toward the cytoplasm, which inhibits the expression of Nrf2 target genes (Salazar et al., 2006). In this study, we observed decreased nuclear Nrf2 and increased cytoplasmic Nrf2 in the hippocampi of the DeS + Veh rats, which indicated an increase in the nuclear export of Nrf2 (Figures 6E,F). Nuclear export of Nrf2 was not observed in the hippocampi of the DeS + Emo rats (Figures 6E,F). Data suggested that emodin inhibited hippocampal GSK3β activation during the development of depression.

## Discussion

**FIGURE 7:**
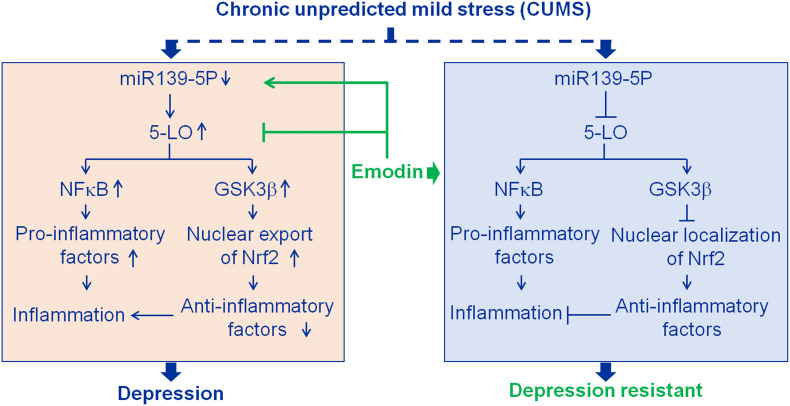
Results summary diagram. Hippocampal inflammation plays an important role in depression. Emodin prevented chronic unpredicted mild stress (CUMS)-induced depression mainly by targeting miR139-5p/5-lipoxygenase (5-LO). Nuclear factor-κB (NF-κB) and glycogen synthase kinase 3β (GSK3β) are the downstream factors of 5-LO. Nuclear factor erythroid 2-related factor 2 (Nrf2), a transcriptional activator of antioxidant genes, down-regulated by activated GSK3β.

In this study, emodin blocked the occurrence of psychosocial stress-induced depression (Figure 7). Significantly up-regulated hippocampal differentially expressed proteins, such as Fga, Fgb, Fgg, Ahsg, Col1a1, and Anxa2, suggested hippocampal inflammation in the development of depression, which was confirmed by the activated microglia and the increased IL-1β, TNF-α, and NF-κB p65 in the hippocampi of the depressed rats. Fg is a marker of inflammation, and increased Fg has been reported to contribute to NF-κB activation (Sulimai and Lominadze, 2020). Fga, Fgb, and Fgg levels were found to be up-regulated in whole blood samples of patients with depression (Wang et al., 2019). Ahsg (also known as fetuin-A), an endogenous ligand for TLR4, has been reported to be involved in lipid-induced inflammation by activating NF-κB (Bryant et al., 2010; Pal et al., 2012; Wang et al., 2012). Elevated serum Ahsg levels were reported in male patients with major depressive disorder (MDD) (Ramsey et al., 2016). Increased hippocampal 5-LO and its major metabolic product, LTB4, suggested the activation of 5-LO. As an inhibitor of 5-LO (Jin et al., 2011), emodin prevented the depression behaviors along with a series of pathological changes in the hippocampus, such as hippocampal neuron and spine loss, microglial activation, increased IL-1β and TNF-α, and the activation of 5-LO and GSK3β. Furthermore, we found that emodin may inhibit 5-LO related inflammation by targeting miR-139-5p. Thus, our study also uncovered a novel role of miR-139-5p and 5-LO in the pathogenesis of depression.

Psychosocial stress is one of the leading factors in the development of depression. When exposed to the same stress conditions, some individuals do not show depressive symptoms, which is called depression resistance. Understanding the mechanism of depression resistance is helpful for preventing the occurrence of depression. In this study, 43.3% of the rats showed significant depression resistance, while 46.67% developed depression after 7 weeks of CUMS exposure. Although previous studies have reported different pathophysiological characteristics between depression and depression-resistant animals (Bisgaard et al., 2007; Henningsen et al., 2012), our results highlighted that 5-LO activation-associated hippocampal inflammation was the key pathological process. When CUMS was exposed, rats presented depression as long as inflammation occurred in their hippocampi. While inflammation did not occur or was inhibited, the rats showed resistance to depression. MiR-139-5p, a neuron-enriched miRNA in the brain, was found to be markedly suppressed in the hippocampus of the depressed rats. It has been demonstrated to attenuate brain damage by regulating neuronal apoptosis and mediating behavioral responses to chronic stress in rats (Qu et al., 2014; Chen et al., 2015). Here, we showed that miR-139-5p specifically bonded to the 3′-UTR of *5-LO* and repressed 5-LO expression. Moreover, emodin prevented a decrease in miR-139-5p and an increase in 5-LO, and effectively ameliorated hippocampal NF-κB p65 upregulation, increased pro-inflammatory factors, and microglial activation in the depression rats. These findings strongly suggest the critical roles of miR-139-5p and 5-LO in the pathogenesis of psychosocial stress-induced neuroinflammation.

Activated GSK-3β has been reported in the brains of patients with depression, and GSK-3β inhibition is a therapeutic target for depression (Beurel et al., 2015; Morlet et al., 2018). Although 5-LO has been shown to be involved in stress-induced activation of GSK3β (Joshi et al., 2014a), the underlying mechanism remains unclear. In this study, GSK3β activation was observed in the hippocampi of the DeS + Veh rats by decreasing the phosphorylation levels of GSK3β at Ser-9. When 5-LO was down-regulated after emodin treatment, GSK3β activity was partially restored. The phosphorylation of GSK3β at Ser-9 is regulated by phosphoinositide 3-kinase/protein kinase B (PI3K/AKT), mitogen-activated protein kinase/extracellular signal-regulated kinase (MAPK/ERK), protein kinase A (PKA), integrin-linked kinase (ILK), calcium/calmodulin-dependent protein kinase 2 (CaMK2), and Wnt signaling (Duda et al., 2020). In contrast, dephosphorylation of Ser-9 by protein phosphatase 1 (PP1), PP2A, and PP2B directly activates GSK3β (Duda et al., 2020). Further exploration of how 5-LO activates GSK3β is of great significance and is needed to understand the key role of inflammation in the occurrence of depression.

Nrf2, a transcription factor, plays a central role in regulating inflammation and regulating the production of antioxidants and antioxidant enzymes (Ramos-Gomez et al., 2001; Rojo et al., 2010; Zhang et al., 2013). It has been reported that ablation of Nrf2 triggered depression-like behaviors related to increase inflammation, and Nrf2 agonists had antidepressant-like effects in an animal model of depression (Martín-de-Saavedra et al., 2013). Under basal conditions, Keap-1 maintains Nrf2 in the cytoplasm by forming the Keap1-Nrf2 complex, and Nrf2 is then ubiquitinated and subsequently degraded by the 26S proteasome (McMahon et al., 2006). When a moderate stimulus appears, Nrf2 is phosphorylated and transferred to the nucleus, which then plays a protective role (Li and Kong, 2009; Lee et al., 2013). GSK3β has been reported to directly phosphorylate Nrf2 and shift the subcellular distribution of Nrf2 toward the cytoplasm, which inhibits the expression of Nrf2 target genes (Salazar et al., 2006) and contributes to the cytoplasmic degradation of Nrf2 (Cuadrado, 2015). In the DeS + Veh rats, increased nuclear export of Nrf2 was observed. Thus, the increased nuclear export of Nrf2 in the hippocampus of depressed rats might be caused by GSK3β activation. GSK3β inhibitors are now being developed as Nrf2 activators (Lastra et al., 2021), suggesting that emodin might act as a potential Nrf2 activator.

Emodin has been reported to inhibit lipopolysaccharide (LPS)-induced microglial activation, as well as protect the synaptic transmission of hippocampal neurons from glutamate excitotoxicity (Gu et al., 2005; Park et al., 2016). Whether emodin can cross the blood–brain barrier is not yet known. A recent study (Taler et al., 2021) and the proteomic data in this study showed hippocampal BBB disruption after CUMS, which promoted emodin to enter the hippocampus conveniently. Additionally, CUMS has been shown to induce inflammation in the liver and pancreas of rats (López-López et al., 2016) and vascular inflammation in rabbits (Lu et al., 2013). Emodin may also antagonize the systemic inflammation induced by CUMS, which requires further experimental confirmation.

Our results are consistent with those of previous studies, which found that pharmacological inhibition of 5-LO ameliorated depression-like behaviors or cognitive impairment (Uz et al., 2008; Luo et al., 2016). In our study, CUMS induced the activation of 5-LO in the DeS + Veh rats. Although the elevated 5-LO levels were not restored to normal levels in the DeS + Emo rats, the emodin-treated DeS rats had normal 5-LO activity. Previous research suggested that stress led to an elevation of 5-LO activity due to the level and translocation of 5-LO (Ford-Hutchinson et al., 1994; Wu et al., 2012). In another study, 5-LO in the nucleus was found to significantly increased in the DeS + Veh rats, while emodin-treated DeS rats had an evaluated 5-LO in the cytoplasm rather than in the nucleus. As mentioned above, decreased miR-139-5p levels in the DeS + Veh rats were reduced by emodin treatment. These findings indicated that emodin not only decreased 5-LO levels by acting on miR-139-5p, but also decreased 5-LO activity by inhibiting its nuclear translocation.

## Conclusion

Taken together, this study demonstrated that emodin blocked the occurrence of CUMS-induced depression by inhibiting 5-LO related inflammation by upregulating miR-139-5p. These results established a key role of miR-139-5p/5-LO in the development of stress-induced depression and provide important evidence that emodin may be a candidate agent for the treatment of depression.

## Data Availability Statement

The datasets presented in this study can be found in online repositories. The names of the repository/repositories and accession number(s) can be found below: ProteomeXchange, via PRIDE partner repository, accession: PXD025773.

## Ethics Statement

The animal study was reviewed and approved by Animal Care and Use Committee at the Huazhong University of Science and Technology.

## Author Contributions

TZ and CY conceived and directed the study, interpreted the results, and wrote the manuscript. TZ and QT designed and performed most of the experiments and analyzed most of the results. JC, L-NN, PZ, X-MW, YS, and B-JQ contributed to the experiments. NQ and QZ provided supervision. TZ, L-NN, NQ, and QT provided funding for experiments. All authors read and approved the final manuscript.

## Conflict of Interest

The authors declare that the research was conducted in the absence of any commercial or financial relationships that could be construed as a potential conflict of interest.

## Publisher’s Note

All claims expressed in this article are solely those of the authors and do not necessarily represent those of their affiliated organizations, or those of the publisher, the editors and the reviewers. Any product that may be evaluated in this article, or claim that may be made by its manufacturer, is not guaranteed or endorsed by the publisher.

## Footnotes

1 http://www.targetscan.org

2 http://www.mirbase.org/

3 http://mirdb.org/
